# Cold Laser Sintering of Medicines: Toward Carbon Neutral Pharmaceutical Printing

**DOI:** 10.1021/acssuschemeng.4c01439

**Published:** 2024-07-16

**Authors:** Moe Elbadawi, Hanxiang Li, Paromita Ghosh, Manal E. Alkahtani, Bingyuan Lu, Abdul W. Basit, Simon Gaisford

**Affiliations:** † School of Biological and Behavioural Sciences, Queen Mary University of London, Mile End Road, London E1 4DQ, United Kingdom; ‡ UCL School of Pharmacy, University College London, 29-39 Brunswick Square, London WC1N 1AX, United Kingdom; § Department of Pharmaceutics, College of Pharmacy, Prince Sattam bin Abdulaziz University, Alkharj 11942, Saudi Arabia

**Keywords:** Additive Manufacturing, Automation, Carbon Neutral, Digital Green Innovation, Digital Technology, Green Engineering, Sustainability

## Abstract

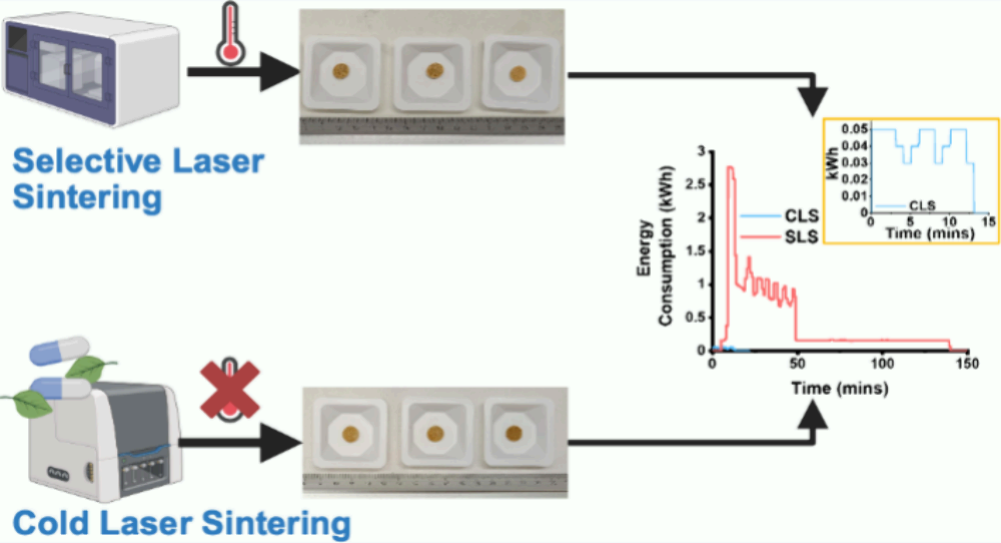

Selective laser sintering (SLS) is an emerging three-dimensional (3D) printing technology that uses a laser to fuse powder particles together, which allows the fabrication of personalized solid dosage forms. It possesses great potential for commercial use. However, a major drawback of SLS is the need to heat the powder bed while printing; this leads to high energy consumption (and hence a large carbon footprint), which may hinder its translation to industry. In this study, the concept of cold laser sintering (CLS) is introduced. In CLS, the aim is to sinter particles without heating the powder bed, where the energy from the laser, alone, is sufficient to fuse adjacent particles. The study demonstrated that a laser power above 1.8 W was sufficient to sinter both KollicoatIR and Eudragit L100-55-based formulations at room temperature. The cold sintering printing process was found to reduce carbon emissions by 99% compared to a commercial SLS printer. The CLS printed formulations possessed characteristics comparable to those made with conventional SLS printing, including a porous microstructure, fast disintegration time, and molecular dispersion of the drug. It was also possible to achieve higher drug loadings than was possible with conventional SLS printing. Increasing the laser power from 1.8 to 3.0 W increased the flexural strength of the printed formulations from 0.6 to 1.6 MPa, concomitantly increasing the disintegration time from 5 to over 300 s. CLS appears to offer a new route to laser-sintered pharmaceuticals that minimizes impact on the environment and is fit for purpose in Industry 5.0.

## Abstract 2

A low-energy consuming laser sintering process that produces medicines on demand.

## Introduction

Selective laser sintering (SLS) is a three-dimensional (3D) printing technology that uses energy from a laser to fuse powder particles together and has been used to fabricate solid dosage forms.^1,2^ While there are a number of emerging 3D printing technologies,^3^ SLS possesses many desirable attributes, including suitability for use with existing pharmaceutical materials, short preprocessing, no need for solvent, and the capability of printing complex geometries. Despite its infancy, SLS has been demonstrated to be a versatile technology capable of fabricating a range of drug delivery systems, including tablets with braille patterns,^4^ intrauterine devices,^5^ drug-loaded synthetic bone grafts,^6^ modular devices,^7^ and complex 3D gyroid geometries.^8^ SLS has also been used to print conductive material,^9^ microfluidic valves,^10^ and shape memory polymers,^11^ all of which are relevant to drug delivery. Beyond pharmaceutics, SLS has been adopted in industrial sectors, such as the aerospace and automotive industries.^12^ Thus, SLS has much potential for industrial application.

SLS takes advantage of the sintering process; adjacent particles fuse together when heated with energy from the laser. The temperature rise often needs to be significant because the particles must soften or melt, but the time spent at high temperature is short as the laser passes quickly over the material.^6,13^ For polymers, the energy provided during SLS allows the polymer chains to first disentangle and then migrate to interlink with the polymer chains in adjacent particles. When the energy is removed, the interlinked chains cool and remain entangled together, hence fusing adjacent particles together. In commercial SLS printers, the energy required to sinter is supplied from two sources; the powder bed, itself, is preheated in the printer, thereby raising it to just below its sintering temperature, before the laser supplies the energy to exceed the sintering threshold in a selective manner.^14,15^ As laser spot sizes in the order of microns are used, SLS can create dosage forms with high resolution.^16,17^ Preheating is intentionally incorporated in SLS to reduce the energy needed from the laser, which is believed to cause internal stresses and thermal deformation.^2^ For polymers, this may affect the mechanical properties of the finished part,^18^ but it is not known if high laser energies are detrimental to pharmaceutical dosage forms.

However, what is known is that the requirement for preheating is a major disadvantage of SLS. For one, it is responsible for the high energy consumption and, in turn, carbon emission recorded for SLS.^19^ In a recent study that examined the carbon emissions of five 3D printing techniques, SLS exhibited the highest carbon emissions ranging from 5 to 50 times more than the other four 3D printing techniques when printing 10 printlets.^19^ The same study revealed that reducing the chamber temperature from 180 to 80 °C was enough to decrease printing emissions by 41%, which is a positive step toward green manufacturing.^19−22^ The second disadvantage of preheating is it prolongs the printing process, thereby requiring a longer lead time compared with other 3D printers.^14^ Furthermore, preheating and subsequent cooling impact the surrounding, unsintered powder bed (also referred to as the powder cake). As a result, the powder cannot be reused and, thus, is wasted.^1,23^ The powder cake is needed as it acts as a support to help the sintered parts maintain their structural integrity during the printing process. Given that environmental requirements are at the forefront of many economies and pharmaceutical manufacturing decisions,^24^ the combination of relatively high carbon emissions, long lead times, and lack of recyclability collectively hinder the translational prospects of pharmaceutical applications of SLS.

As companies and broader economies are committing to carbon neutrality by 2050, minimizing the carbon emission and resource waste from SLS printing will ensure the technology remains fit for purpose in the Industry 5.0 framework.^25,26^ Recent publications in SLS continue to use high surface and chamber temperatures ranging between 70 and 150 °C.^27^ Furthermore, to the best of our knowledge, the energy consumption between traditional SLS and temperature-free sintering has not been studied. To date, the sintering of pharmaceutical formulations at room temperature and their energy consumption have not been disclosed. This leaves a gap in scientific knowledge regarding the environmental efficiency of temperature-free sintering and how it compares to conventional SLS printing.

To that end, we explored the potential of sintering pharmaceutical formulations without the need for preheating the powder bed in order to improve the sustainability of SLS for fabricating medicines. We demonstrate that sintering can be solely achieved by the laser, thereby eliminating the need for both preheating and *in situ* heating provided that the laser configuration is appropriate. We hypothesize that the polymer chains do not distinguish between the energy supplied by the laser or from heating elements typically found in commercial laser printers. As long as the energy from the laser is sufficient, it will facilitate the entanglement of polymer chains in adjacent particles, thereby allowing sintering to occur. A comparison of energy consumption between the new processing technique, which we refer to as “cold laser sintering” (CLS), and a commercial SLS printer was conducted to elucidate the sustainability prospect of the new platform. The term “cold” was selected to maintain consistency with other green innovation technologies that achieve processability at considerably low temperatures in what otherwise would have been a high-temperature processes, thereby disrupting the manufacturing status quo.^28,29^ Furthermore, we characterized the CLS-printed products with respect to their mechanical, morphological, chemical structural, and dissolution characteristics by primarily examining the effect of the laser power. The objective here was to compare the results to those of SLS-printed products and evaluate the feasibility of CLS as a new method.

## Experimental Procedure

### Materials

Table 1 lists the polymers, active pharmaceutical ingredients (APIs), and sintering agent used for this study and their corresponding suppliers. All APIs were of United States Pharmacopeia (USP) grade.

**Table 1:** Excipients and APIs and Their Corresponding Suppliers

	material	supplier
polymers	Eudragit L100–55	Evonik, UK
	KollicoatIR	BASF, UK
	Kollidon VA64	BASF, UK
	Plasdone S-630	Ashland, UK
	ParteckMXP	Sigma-Aldrich, UK
API	paracetamol	Sigma-Aldrich, UK
	aspirin	Sigma-Aldrich, UK
	ibuprofen	Sigma-Aldrich, UK
sintering agent	Candurin Gold Sheen	Azelis, UK

### CLS and SLS Printing

**Figure 1:**
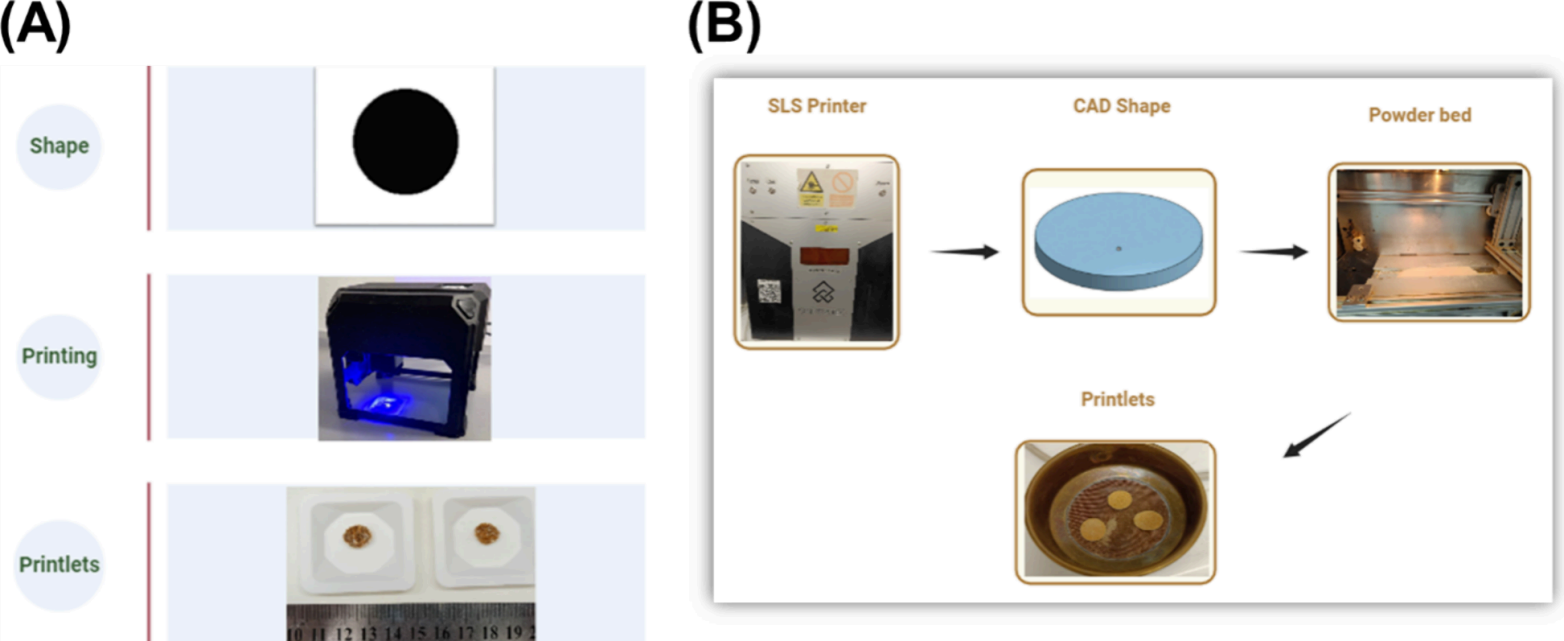
(A) CLS and (B) commercial SLS printing steps.

Pharmaceutical formulations were first individually sieved through a mesh of 150 μm before being mixed with a pestle and mortar until a homogeneous mixture was obtained. CLS was achieved using a commercially available laser engraver (K4 Laser Engraver, HomdMarket, Guangzhou Gesan Network Technology Co Ltd., China) that has a blue diode laser with a power of 3.0 W, a wavelength of 450 nm, and a spot size of 0.05 mm. The laser power was adjustable through the engraver’s software (K4 software v2.7). Five grams of the powder mixture was poured onto a weighing boat that was then placed beneath the laser (Figure 1). Prints were designed using Microsoft Paint, exported as. png file, and were uploaded to the engraver’s software. The software was also used to control the printer, including the key parameters of laser power (0–100%) and laser depth (0–100%). Once printed, samples were removed using a spatula, and the powder was manually replenished. Table 2 lists the formulations tested by the CLS.

**Table 2:** Print Polymer and API Composition as a Function of w/w %^a^

formulation	KollicoatIR	Eudragit L100-55	ParteckMXP	Kollidon VA64	Plasdone S-630	Para	Asp	Ibu
F1	92					5		
F2	62					35		
F3	57					40		
F4	57						40	
F5	57							40
F6		57				40		
F7		57					40	
F8		57						40
F9			92			5		
F10				92		5		
F11					92	5		

a All formulations contained 3 w/w % Candurin. Para, paracetamol; Asp, aspirin; Ibu, ibuprofen.

A commercial SLS printer was used to compare the performance of the CLS process with that of conventional SLS printing. For SLS printing, 20 g of powder was transferred to the SLS printer (Sintratec Kit, AG, Switzerland). The printer has a blue diode laser with a power of 2.3 W and a wavelength of 445 nm. Tablets were designed using computer-aided design (CAD) software (Onshape, PTC, USA) with a diameter of 10 mm and a thickness of 1 mm to match the dimensions of the CLS prints. The CAD models were then exported as an .stl file and uploaded on the printer’s software (Sintratec Central software, v2.5.1). SLS printing was performed at a chamber temperature of 90 °C, surface temperature of 110 °C, and laser scanning speed of 90 mm/s. Three samples were printed per printing run. Formulation F1 was used for the comparison.

### Characterization

#### Physical Properties

The diameter and thickness of the prints were measured by using a digital Vernier caliper. The weight was also determined using a scale (XS105 Dual Range, Mettler Toledo, Switzerland).

#### X-ray Diffraction (XRD)

The XRD patterns were obtained with a Rigaku MiniFlex 600 (Rigaku, Wilmington, MA, USA) equipped with a Cu Kα X199 ray source (λ = 1.5418 Å). The intensity and voltage applied were 15 mA and 40 kV, respectively. Samples were scanned between 2θ = 3–60° with a stepwise size of 0.02° at a speed of 10°/min.

#### Differential Scanning Calorimetry (DSC)

Powdered samples (5–10 mg) were analyzed using Tzero pans (TA Instruments, DE, USA). A Q2000 DSC (TA Instruments, DE, USA) equipped with an autosampler and nitrogen for both cooling and purging (50 mL/min) was used to determine the thermal profiles of all samples. Following initial acclimatization to 40 °C, the temperature was raised to 200 °C at a heating rate of 10 °C/min. For printed samples, a pestle and mortar were used to grind the prints into a powder.

#### Attenuated Total Reflectance Fourier Transform Infrared Spectroscopy (ATR-FTIR)

The vibrational bands of the samples were obtained using a Spectrum 100 spectrometer (PerkinElmer, CT, USA). Similar to DSC characterization, the raw materials were added as-sieved, whereas prints were first ground into powder using a pestle and mortar. Samples were added onto the crystal, and the force of the arm of the universal attenuated total reflectance accessory (UATR) was set to 130. The spectral data was analyzed with the Essential FT-IR software (V3.10.016, Operant LLC, WI, USA). Data was collected over the wavenumber range from 4000 to 650 cm^–1^ with a resolution of 2 cm^–1^ and 8 scans obtained per sample.

#### Drug Loading

Samples were placed in separate volumetric flasks with 250 mL of distilled water and under magnetic stirring until complete dissolution. Samples of the solution were then withdrawn using a syringe equipped with a 0.22 μM filter (Millipore Ltd., Ireland), and the concentration was determined using a UV–vis spectrometer (Cary 100, Agilent Technologies, UK) at 247 nm.

#### Dissolution Study

A dissolution bath was used to determine the release profile of the CLS prints. A USP II dissolution apparatus (PTWS 100, Pharmatest, Hainburg, Germany) was filled with 900 mL of 0.1 M HCL (pH 1.2) to simulate gastric conditions. The paddle speed was set to 500 rpm, and the temperature was 37 ± 0.5 °C. Five mL dissolution samples were withdrawn at predefined times (min). The dissolution samples were then filtered through a 0.22 μm PTFE filter (Merck Millipore Ltd., Ireland), and the concentration was calculated using a UV–vis spectrometer (Cary 100, Agilent Technologies, UK). A total of three repeats was used per group.

#### Disintegration Test

The Petri dish method was used to determine the disintegration time of CLS prints. A 100 mm diameter glass Petri dish containing 20 mL of distilled water was maintained at 37 ± 0.5 °C. CLS tablets (10 mm × 1 mm) were placed on the Petri dish, and the time needed for total disintegration was recorded. Three prints were tested for each group.

#### Energy Consumption

Energy consumptions of both the CLS and SLS printer were measured with an energy meter (Electrocorder AL-2VA, Acksen Ltd., UK) with a 5 s sampling rate.

#### Mechanical Testing

Friability of the CLS tablets was determined using a friability tester (Erweka type TAR 10, Erweka GmbH, Heusenstamm, Germany). Tablets were added to the drum of the tester and rubbed at 25 rpm for 4 min. The tablets were weighed before and after the test, and their weight loss as a percentage was recorded. In addition, a dynamic mechanical analyzer (DMA) was used to calculate the flexural properties of rectangular prints (20 mm × 10 mm). Samples were placed in the DMA (Q800, TA Instruments, USA) equipped with the three-point bending clamp (submersion option).^30^ Measurements were performed under ambient conditions and with the furnace open. Samples were analyzed at a ramp rate of 0.5 N/min. Three repeats were used for each group where their length, width, and thickness were measured using a digital Vernier calliper.

#### Microstructural Analysis

Scanning electron microscopy (SEM) was used to image the sample microstructure. Samples were adhered onto a carbon adhesive attached to an aluminum stub. Samples were gold-coated using a sputter coater (Quorum Q150RS plus, Lamda Photometric, UK) for 120 s. Samples were then inserted into the scanning electron microscope (Phenom Pro Desktop, Thermo Fisher Scientific, UK) and imaged at 5 kV.

## Results

We began our study by attempting to print the formulations from the seminal work by Fina et al.^31^ using the commercial SLS printer but at room temperature (formulations F1–F4 from Table 2). However, no evidence of sintering was observed in which the powder cake remained in its particulate form. In contrast, CLS was successfully found to sinter the same formulations into a solid dosage form, which confirmed our hypothesis that the energy supplied by the laser, alone, was sufficient to sinter the powder. The findings emphasize the limitation of the commercial printer’s laser configuration, including laser power, for sintering at room temperature.

**Figure 2:**
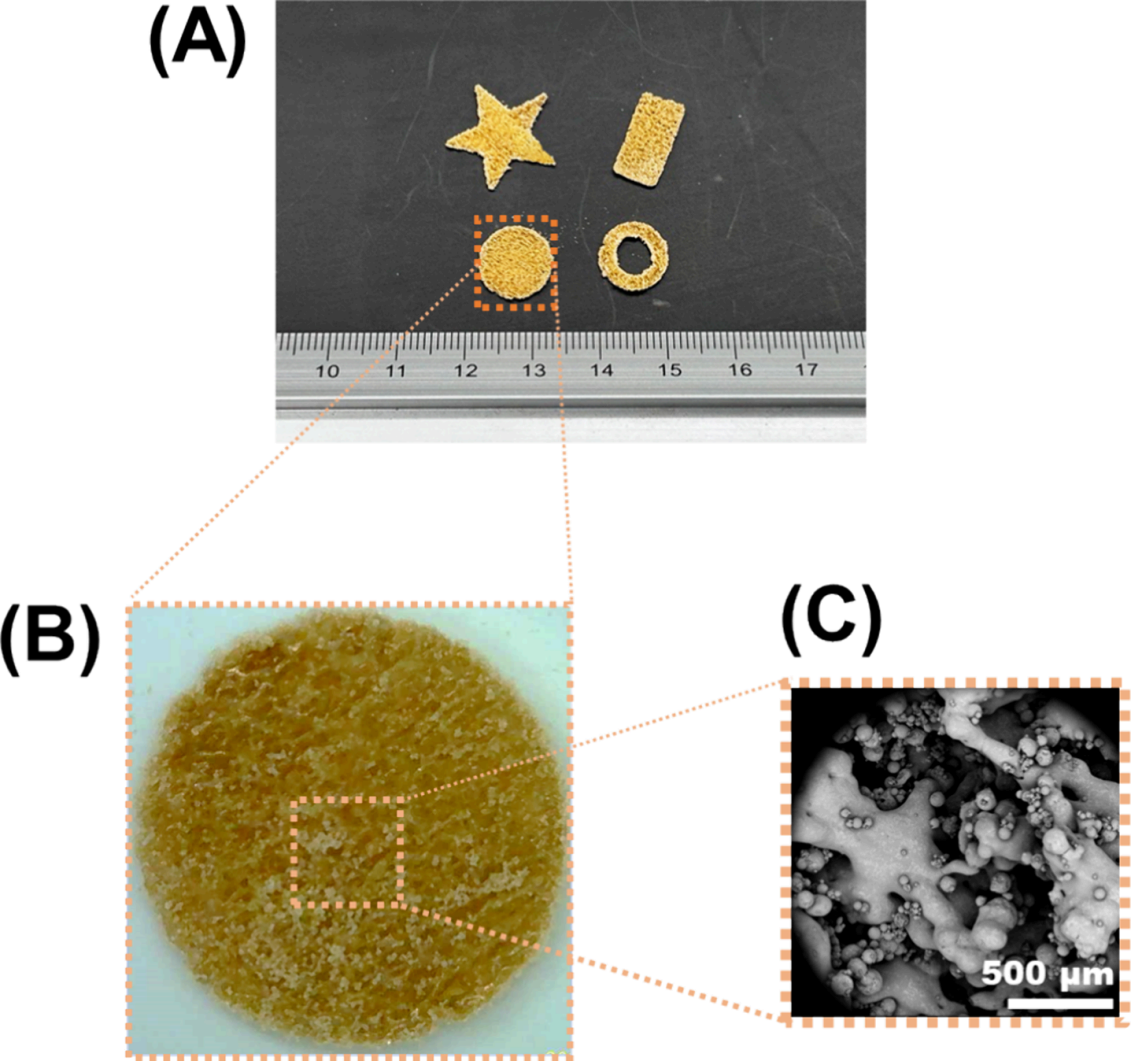
Multiscale imaging of formulation F1 prints processed by CLS. (A) Different geometries were printed by CLS. (B) Digital microscope image of the tablet print. (C) SEM image of (B).

Furthermore, different shapes were sintered using formulation F1 to elucidate the potential of CLS for fabricating different geometric features (Figure 2A). Visual inspection revealed a rough surface (Figure 2B) with SEM imaging highlighting typical sintering features, such as pores and sintering necks (Figure 2C). In addition, the versatility of CLS was tested by printing the remaining F5–F11 formulations composed of other pharmaceutical grade polymers, which were Eudragit L100-55, Parteck MXP, Kollidon VA64, and Plasdone S-630. The results revealed that CLS was capable of sintering these formulations, thus demonstrating its compatibility with five pharmaceutical-grade polymers. The results for Eudragit will be discussed below, whereas the evidence for Parteck, Kollidon, and Plasdone can be found in the Supporting Information (Figures S1 and S2).

**Figure 3:**
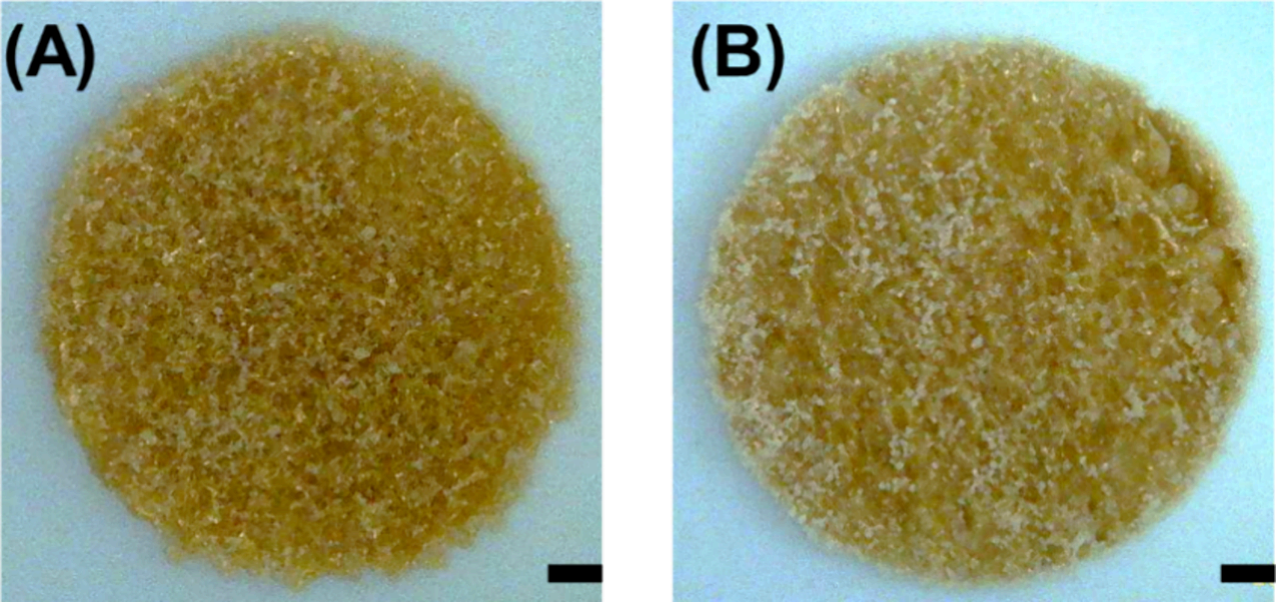
Digital microscopy image of formulation F1 processed by (A) CLS and (B) SLS prints. The images reveal a similar surface morphology between the two processed printlets (scale = 1 mm).

**Figure 4:**
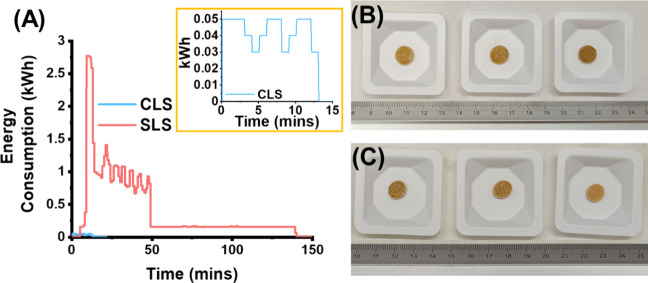
Panel (A) presents the energy consumption results for both SLS and CLS for printing three printlets of 10 mm × 1 mm dimensions using formulation F1. The inset provides a zoomed-in view of the CLS results. The three printlets of (B) CLS and (C) SLS are also presented.

We then compared the energy consumption between CLS and the commercial SLS printer by printing three printlets for each technology from formulation F1. For the SLS printer, we applied the same parameters from the seminal work by Fina et al.,^31^ which were a chamber and surface temperature of 90 and 110 °C, respectively (Figure 3). This allowed us to determine whether CLS was energy efficient. The total energy required for SLS was 1.01 kWh. That included the following steps: preheating, building, and cooling (Figure 4A).^32^ For CLS, the total energy required was 0.01 kWh. Thus, CLS was able to reduce the energy consumption by 99% compared with SLS. The total time needed to print three dosage forms by SLS was 138 min, encompassing the preheating, sintering, and cooling stages. In contrast, CLS required 13 min to fabricate three printlets. These findings highlight that a more energy-efficient laser sintering process is attainable compared to conventional SLS printing.

### Effect of Laser Power

Once it was confirmed that CLS was capable of fabricating solid dosage forms, the effect of the laser power was investigated. The commercial printer used by Fina et al. does not provide the opportunity to control the laser power, and thus, there was an opportunity to understand the effect of the laser power on formulation F1.

**Figure 5:**
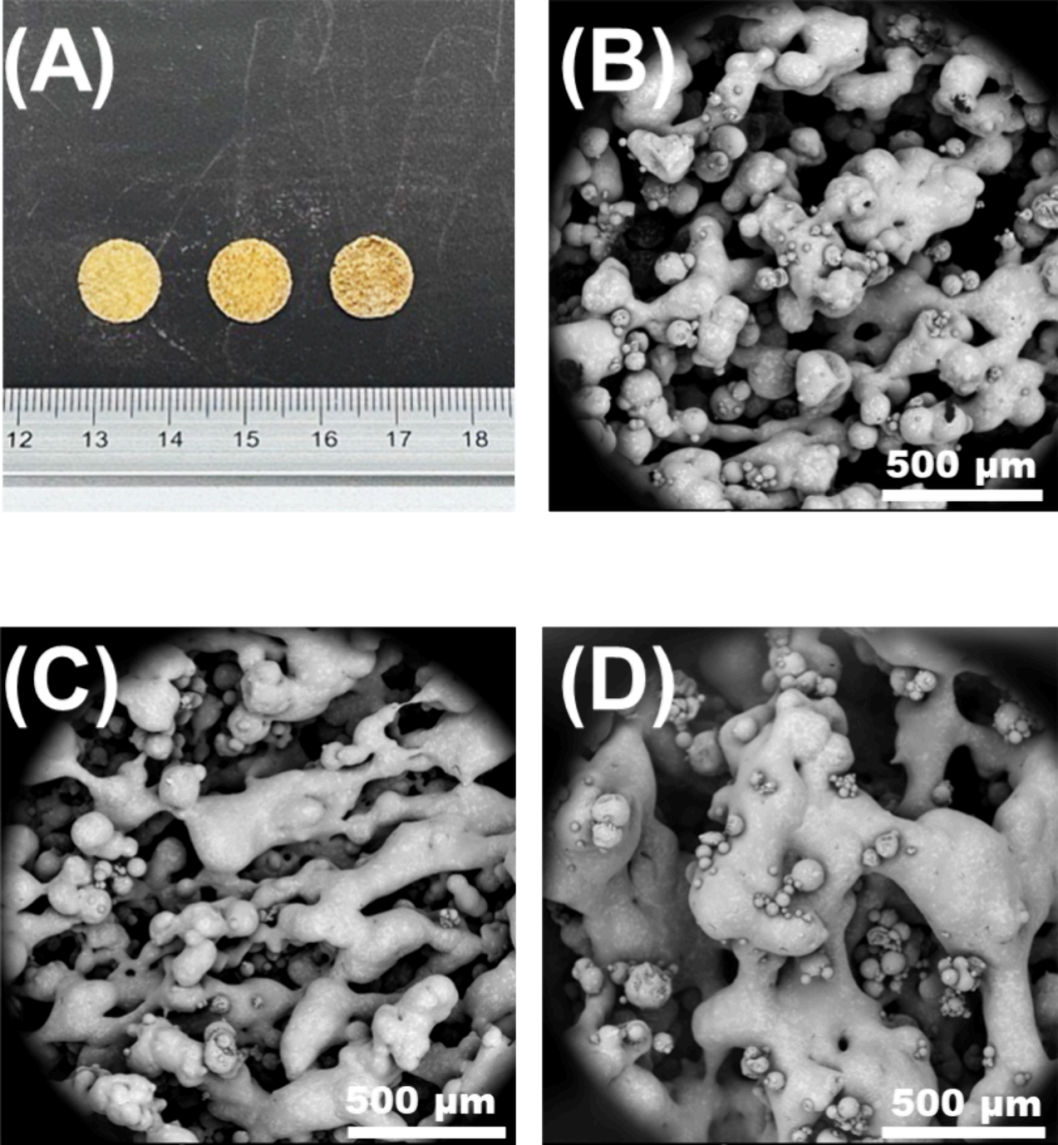
(A) From left to right, printlets of formulation F1 processed at L60, L80, and L100. SEM images were taken of each printlet, and the micrographs for (B) L60, (C) L80, and (D) L100 are presented.

**Figure 6:**
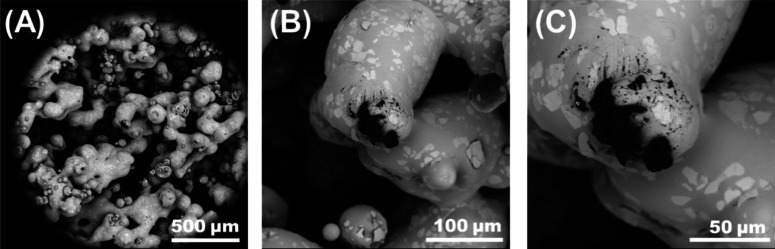
SEM micrographs of formulation F1 processed at L60 at different microscales. Samples processed at this laser power exhibited “tears,” which suggest low cohesion in the material at the microscale, which causes particles to “fall off.”

Three different laser powers were tested: 40%, 60%, 80%, and 100%—referred to as L40, L60, L80, and L100 with L100 being equivalent to 3.0 W. It was found that laser powers above L60 were capable of sintering the formulation, whereas L40 did not. Several features were observed with an increasing laser power. For one, the printlets color changed with the color become a deeper shade of gold (Figure 5A). Microstructural analysis revealed that the porosity decreased with increasing laser power (Figure 5B–D). Moreover, the sintering necks were found to be thicker at L100. In addition to exhibiting comparatively high porosity, samples sintered at L60 displayed evidence of tears (Figure 6), possibly as a result of partial sintering, which caused particles to fall off.

**Figure 7:**
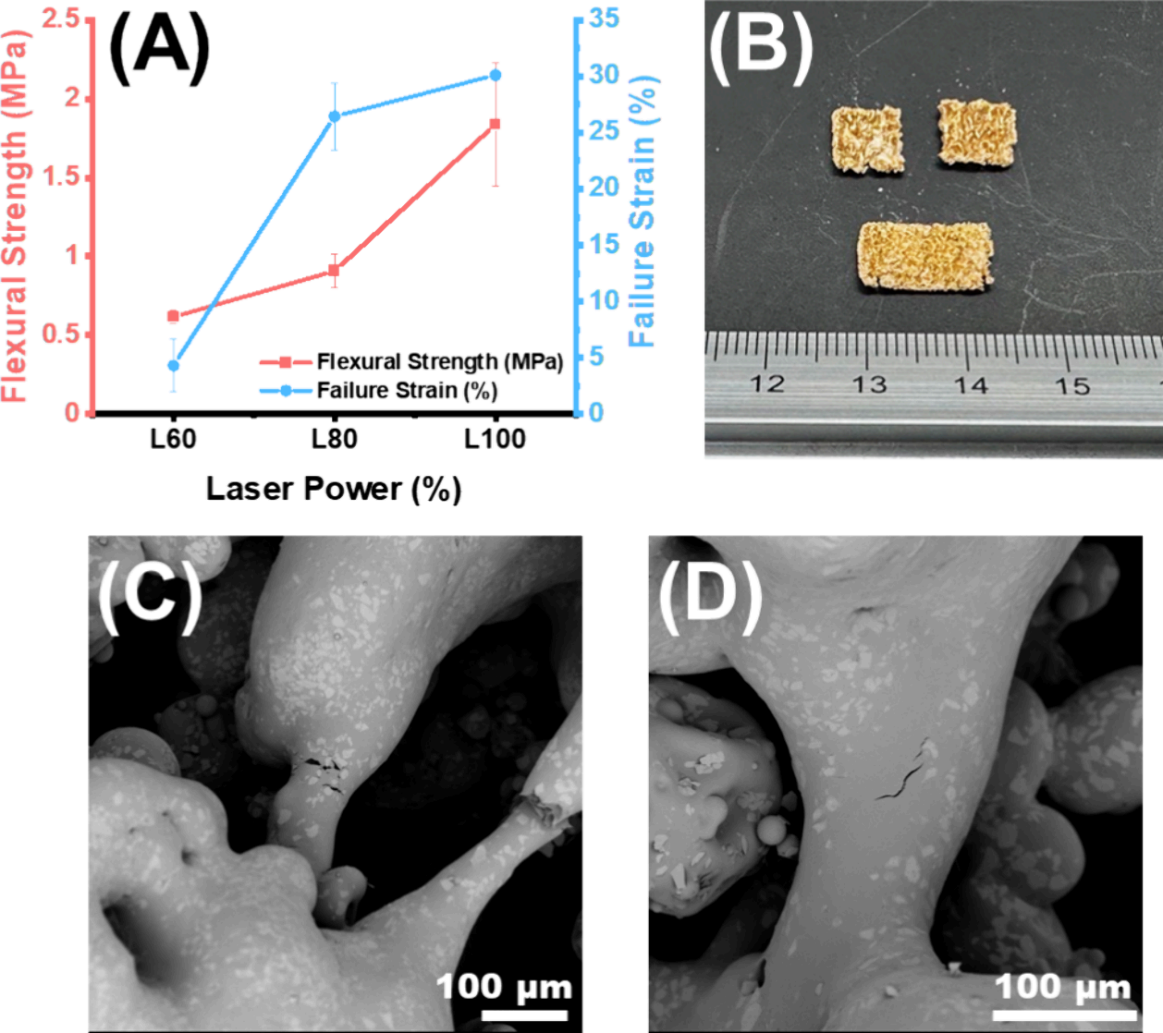
(A) Flexural mechanical properties of formulation F1 processed at different laser powers (*n* = 3). (B) Photograph images of L60 (top) and L100 (bottom) following a flexural test. SEM micrograph images of (C) L60 and (D) L100 following a flexural test.

Mechanically, the samples were found to vary significantly in their flexural properties (Figure 7A). The flexural strength increased from 0.62 ± 0.04 MPa at L60 to 0.91 ± 0.10 MPa at L80 and to 1.84 ± 0.39 MPa when sintered at L100. A similar trend was observed for the failure strain, whereby increasing the laser power resulted in an increase in the fracture resistance. In fact, samples sintered at L100 did not fracture completely like samples sintered at a lower laser power (Figure 7B). Collectively, the flexural analysis revealed that increasing the laser power improved both the strength and the ductility of the sintered samples. The fractured samples were imaged using SEM to reveal potential causes of failure at the microstructural level. SEM revealed that fracturing occurred on the sintering necks, thereby inferring that they were structurally the weakest point. Furthermore, thicker sintering necks were more resistant to fracturing (Figure 7C,D). Friability of the F1 formulation under different laser conditions was also recorded. The results were 1.87%, 0.73%, and 0.23% for L60, L80, and L100. Thus, the latter two complied with the US pharmacopeia requirements for uncoated tablets as they were less than 1%, thereby making them suitable for handling and packing.^33^

**Figure 8:**
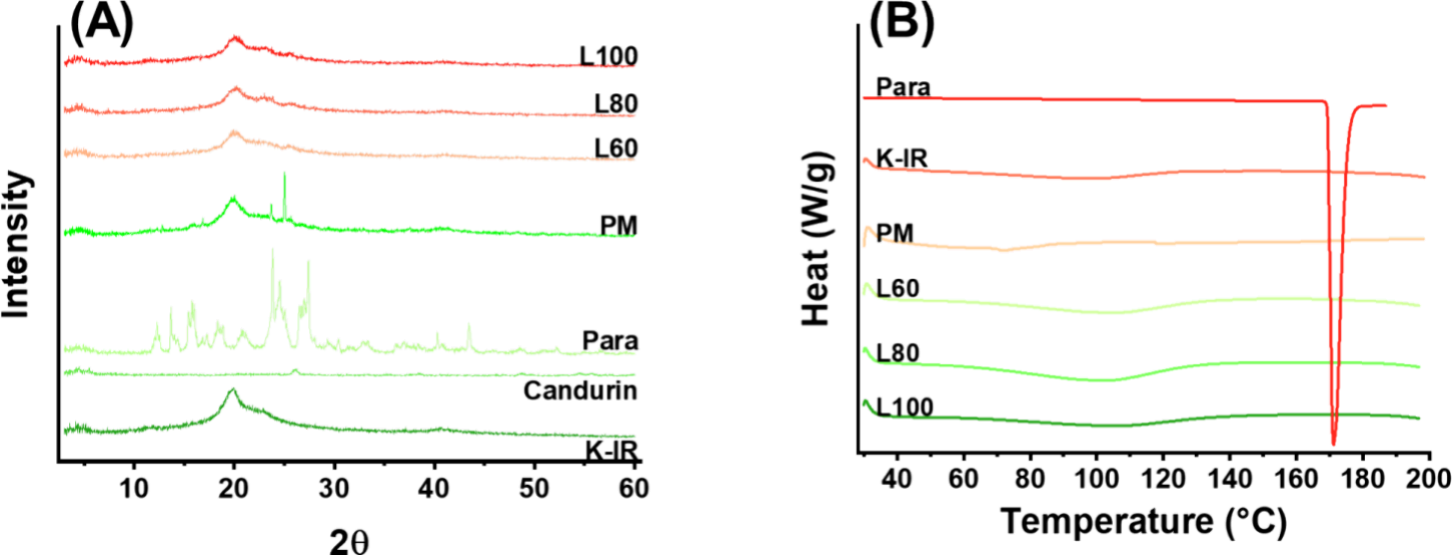
Physicochemical analysis of the raw materials, formulation F1 powder mixture (PM), and samples processed at different laser powers using (A) XRD and (B) DSC. Both XRD and DSC suggest that CLS amorphised paracetamol.

**Figure 9:**
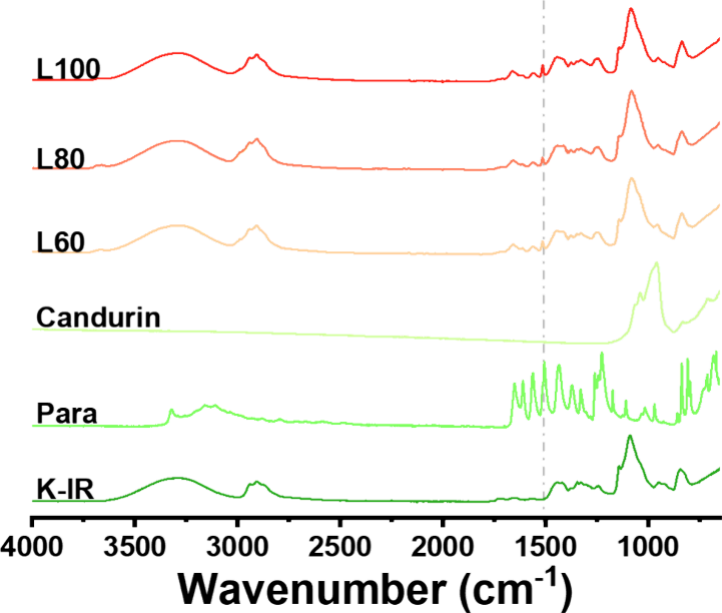
ATR-FTIR results of the raw materials and formulation F1 processed at different laser powers. The dashed gray line highlights peaks unique to paracetamol that were also found in formulations processed by CLS. The data suggest that paracetamol remained intact.

Physicochemical analyses were conducted before and after CLS processing to reveal the effect of the laser on the formulation. XRD analysis of raw paracetamol revealed its distinct crystalline structure (Figure 8A). Some of these peaks were still present in the physical mixture; however, they diminished following CLS processing at all three laser powers. This suggests that paracetamol was amorphised during printing. DSC analysis of the printlets at all three laser powers revealed the absence of melting at 170 °C, which was present in the raw paracetamol (Figure 8B). For further clarity, ATR-FTIR was performed since it is capable of detecting amorphous materials. The results revealed that the KollicoatIR peaks^34^ dominated the FTIR spectra for formulations processed at L60, L80, and L100. The fingerprinting region (1500–650 cm^–1^) is a region that uniquely identifies materials, but as can be seen in Figure 9, between 1500 and 650 cm^–1^, all three raw materials’ vibrational bands overlap. However, raw paracetamol contains peaks between 1644 and 1507 cm^–1^^35^ that are not present in either KollicoatIR or Candurin, which were also present in L60, L80, and L100 formulations. Thus, the results suggest that paracetamol is present in the printlets in an amorphous state.

A drug loading efficiency analysis was performed to verify the presence of paracetamol within the printlets using UV–vis spectroscopy; drug loadings varied between 99.68 ± 4.03% and 105.25 ± 1.71% (Table 3). Thus, it is clear that paracetamol remained intact at all three laser powers. In addition, the disintegration time was also recorded because fast disintegration times are characteristic of SLS prints.^36^ Printlets produced at L60 were found to dissolve in 5.21 ± 0.76 s, while at L80 they required 53.39 ± 6.91 s (Table 3). However, samples sintered at L100 showed no signs of disintegrating, and the tests were stopped after 300 s. Therefore, while the laser power did not affect drug loading, it did affect the disintegration time.

**Table 3:** Drug Loading Efficiency and Disintegration Time for Formulation F1 Processed by CLS at Different Laser Powers (*n* = 3)

sample	loading efficiency (%)	disintegration time (s)	mass (mg)
L60	99.68 ± 4.03	5.21 ± 0.76	20.9 ± 1.45
L80	105.25 ± 1.71	53.39 ± 6.91	27.90 ± 1.51
L100	103.38 ± 0.14	>300	37.40 ± 1.35

**Figure 10:**
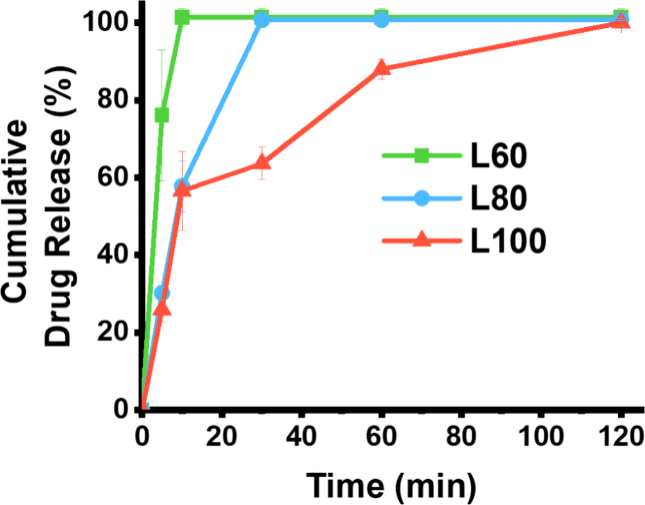
Paracetamol release profile for formulation F1 processed by CLS at different laser powers (*n* = 3).

Drug dissolution was performed to determine the release profile of the printlets as a function of the laser power (Figure 10). Prints processed at L60 were found to achieve 100% release by 10 min, whereas 100% release for prints processed at L80 required 30 min. Prints processed by the highest laser power of L100 required 120 min to achieve 100% release, which is significantly longer than formulations processed at either L60 or L80. Therefore, increasing the laser power reduced the rate of drug release.

### Effect of Drug Loading

**Figure 11:**
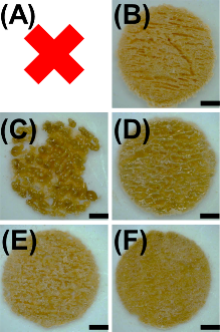
Digital microscope images of both (A,C,E) KollicoatIR and (B,D,F) Eudragit L100-55 formulations processed by CLS at 40 w/w % drug loading using (A,B) paracetamol, (C,D) aspirin, and (E,F) ibuprofen. Note that KollicoatIR containing 40 w/w % paracetamol could not be printed.

In their seminal study, Fina et al.^31^ were able to record a maximum drug loading of 35 w/w % using the KollicoatIR formulations. The authors tested Eudragit L100-55 and also recorded a maximum of 35 w/w % drug loading. Using the CLS, the same was also observed, whereby the maximum paracetamol loading was 35 w/w % for both polymers. At 40 w/w % paracetamol, KollicoatIR formulations could not sinter (Figure 11A), whereas Eudragit L100-55 presented with large fractures (Figure 11B). As sintering is a function of formulation thermal properties, we tested the same composition but replaced paracetamol with either aspirin or ibuprofen. Compared with paracetamol, both aspirin and ibuprofen have lower melting points and, thus, are potentially more amenable to cold sintering. Using 40 w/w % aspirin, the KollicoatIR formulation began to partially sinter (Figure 11C), whereas the Eudragit L100-55 formulation sintered and was free of fractures (Figure 11D). For using 40 w/w % ibuprofen, both formulations successfully sintered (Figure 11E,F). Thus, the results highlight that the drug melting point can affect sintering.

## Discussion

The study was conceived following the recent findings that SLS, despite its many advantages, is energy-intensive and potentially more environmentally harmful than other 3D printing technologies.^19^ There is a pressing need to pursue digital green innovations, which will not only add economic value to industries who utilize such innovation^37^ but also help to improve planetary health. Herein, we confirmed that solid dosage forms *can* be fabricated with CLS without the need for pre- or *in situ* heating of the powder. As expected, CLS was found to be considerably less energy-demanding compared with a commercial SLS printer (Figure 4A). Consequently, CLS was comparable in its carbon emission to other 3D printing techniques,^19,20^ which means it offers a sustainable alternative to SLS for producing solid dosage forms. Potentially, this could lead to cost savings in the long run because of the reduced energy consumption. While more work is needed, the long-term vision will be the development of an industrial CLS device that 3D prints medicines while aligning with global sustainability goals.

Second, precluding the need for heating means that the physicochemical properties of the powder bed can be preserved. The powder bed acts as a support for both SLS and CLS, and any changes to its properties will compromise the stability, efficacy, and safety of the final product. Consequently, the unused powder will have to be discarded. Thus, by avoiding heating of the entire powder bed, any unused powder can potentially be recycled. This not only reduces material wastage but also contributes to the optimization of resources. By allowing for reuse of the powder cake, the overall consumption of raw materials is reduced, thereby leading to further cost savings. Moreover, recycling minimizes the environmental footprint associated with sourcing and processing new materials. As such, the capability of recycling the powder bed amplifies the sustainability benefits of the CLS process, which makes it an even more attractive option for industries focused on sustainable production methods.

This is the first study to compare the energy consumption of both SLS and CLS, and more work is needed to realize their translational potential, especially within the emerging Industry 5.0 framework.^25,38^ It is understood that manufacturing enterprises are interested in green innovation, but currently, their discussions remain at the theoretical level. Hence, it is anticipated that the findings of this study will help to provide a framework for achieving their green innovation goals.^37^ The present findings compliment previous work where there is a growing body of literature seeking to achieve green innovation in 3D printing. Beyond drug delivery, green innovation for 3D printing has explored the possibility of both recycling and upcycling feedstocks.^39^ Other research has explored the potential of using 3D printing materials that can sequester carbon from the environment, thereby minimizing its impact.^40^ Studies into the use of artificial intelligence (AI) have also been investigated, whereby AI can minimize the wasteful practice of trial-and-error and for preventative maintenance.^41^ Furthermore, SLS has been said to possess the potential to be an environmentally benign technology for rapid prototyping compared with traditional processes.^42^ Thus, it is anticipated that these green innovations—hardware, formulation, and AI input—will converge to help ensure pharmaceutical 3D printing is environmentally sustainable.^22,43^

The final aim will be to realize the potential of CLS as a robust industrial pharmaceutical fabrication technique. However, its versatility extends beyond just large-scale production. CLS could also serve as an invaluable tool for rapid screening applications in the laboratory. Removing heating elements can reduce the size of the printer, which means the technology has the potential to be compact and require smaller amounts of powder. This, in turn, translates to cost savings and efficient utilization of resources. Additionally, the inherent efficiency of the CLS technique means that the process is expedited, thereby allowing for faster lead times. This rapidity is particularly beneficial when quick assessments or iterative testing are needed, which ensures that potential issues or variations in material properties can be identified and addressed promptly. As such, industries looking for both production and prototype solutions might find CLS to be a twofold benefit meeting both their fabrication and rapid screening needs.

Moreover, a compact printer will be more likely to be adopted in clinics because of its space-saving design and ease of integration into existing infrastructures. Such a compact footprint allows clinics, even those with limited space, to utilize the technology without the need for extensive modifications or renovations. This not only reduces the barriers to adoption but also ensures that clinics can rapidly deploy and benefit from the technology, thereby enhancing their service offerings and improving patient care outcomes.

As expected, altering the laser power was found to alter several features of the final product. Interestingly, altering the laser power was found to produce ductile/flexible films, which is unheard of because SLS has been reported to produce brittle dosage forms.^44,45^ Previous work in SLS of engineering polymers has also reported that samples become less brittle with increasing laser power.^45,46^ The reason for this is because wider sintering necks improve the overall toughness of the product. Thus, the study alludes to the possibility of producing flexible films, which will further expand the utility of CLS, as flexible films are desirable.^47^ The flexural strength was comparable to previous work investigating 3D-printed oral dosage forms where 3D-printed chocolate dosage forms were found to have a flexural strength of 0.75 ± 0.09 MPa.^48^

Aside from mechanical properties, this study demonstrated that the dosage forms made by CLS possessed desirable features seen in those fabricated by commercial SLS printers, including API amorphization, friability, fast disintegration times, and fast drug release.^31,36^ Further research is, indeed, warranted. The immediate goal is to scale up the technology to ascertain its capability to fabricate 3D structures, thereby broadening its range of applications. It is evident that CLS holds significant promise as a laser fabrication technology perfectly aligned with the principles of Industry 5.0 and characterized by human-centricity and sustainability. The realization of this potential hinges on demonstrating CLS’s ability to produce personalized dosage forms and its commitment to maintaining a minimal environmental footprint.

## Conclusion

The study set out to determine the feasibility of cold laser sintering formulations previously fabricated by using a commercial SLS printer. It was found that the energy from the laser was sufficient to sinter KollicoatIR, Eudragit L100-55, Plasdone S-630, Parteck MXP, and Kollidon VA64 formulations requiring a minimum laser power of 1.8 W. Further increases to the laser power were found to reduce porosity, increase the flexural mechanical properties, and reduce the disintegration and drug release properties of the final solid dosage product. The energy consumption, and by extension carbon emission, for printing three printlets using CLS with the highest laser power (3.0 W) was 99% less than that needed for a commercial SLS printer to also print three printlets. Thus, the study demonstrated the potential for redefining laser sintering that is fit for purpose in Industry 5.0. The findings of this study are encouraging, and future work will seek to scale-up the technology, with the long-term vision being an industrial CLS manufacturing process.

## Data Availability

The data set used and/or analyzed during the current study is available from the corresponding author on reasonable request.

## Supporting Information Available

Photographic images of the ParteckMXP (F9), KollidonVA64 (F10), and Plasdone S-630 (F11) printlets and their corresponding DSC and XRD data of the raw materials, physical mixture, and final print (PDF)
