# Supplementary material for: Cold Laser Sintering of Medicines: Toward Carbon Neutral Pharmaceutical Printing
Source: ACS Sustain Chem Eng. 2024 Jul 16;12(30):11155–66. doi: 10.1021/acssuschemeng.4c01439 (PMC11289754; doi:10.1021/acssuschemeng.4c01439)
Supplement: Supplementary file 1 — sc4c01439_si_001.pdf [file sc4c01439_si_001.pdf]

Supporting Information for

Cold Laser Sintering of Medicines: Towards Carbon Neutral Pharmaceutical Printing

Moe Elbadawi[1], Hanxiang Li[2], Paromita Ghosh[2], Manal E. Alkahtani[2,3], Bingyuan Lu[2], Abdul W. Basit[2] and Simon Gaisford[2]*

[1]School of Biological and Behavioural Sciences, Queen Mary University of London, Mile End Road, London E1 4DQ, UK.

[2]UCL School of Pharmacy, University College London, 29-39 Brunswick Square, London WC1N 1AX, UK.

[3] Department of Pharmaceutics, College of Pharmacy, Prince Sattam bin Abdulaziz University, Alkharj 11942, Saudi Arabia.

* Correspondence: s.gaisford@ucl.ac.uk

Supporting Information contains:

Number of Pages: 2

Number of Figures: 2

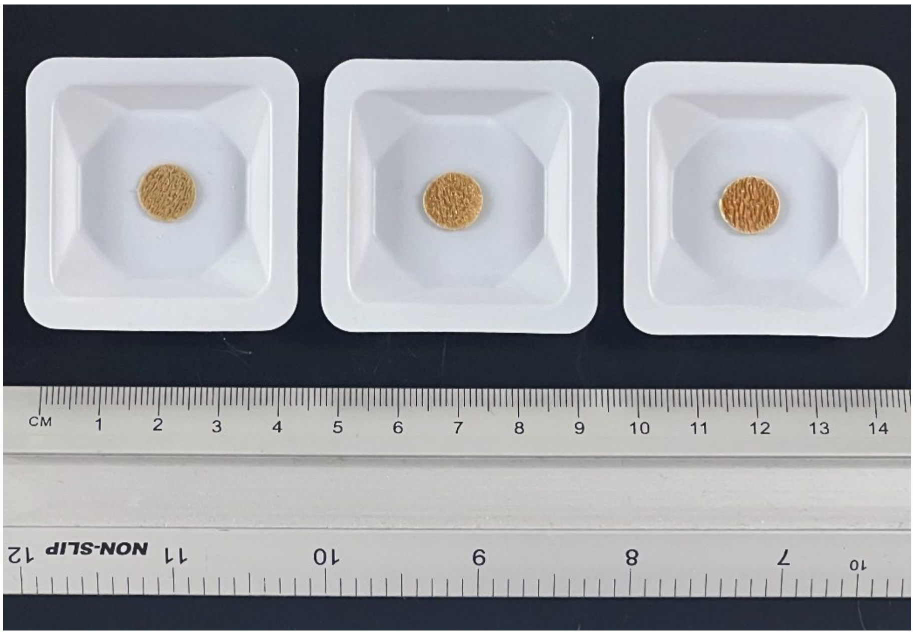

Figure S1. *Printlets using Parteck®MXP (F9), Kollidon®VA64 (F10) and Plasdone™ S-630 (F11).*

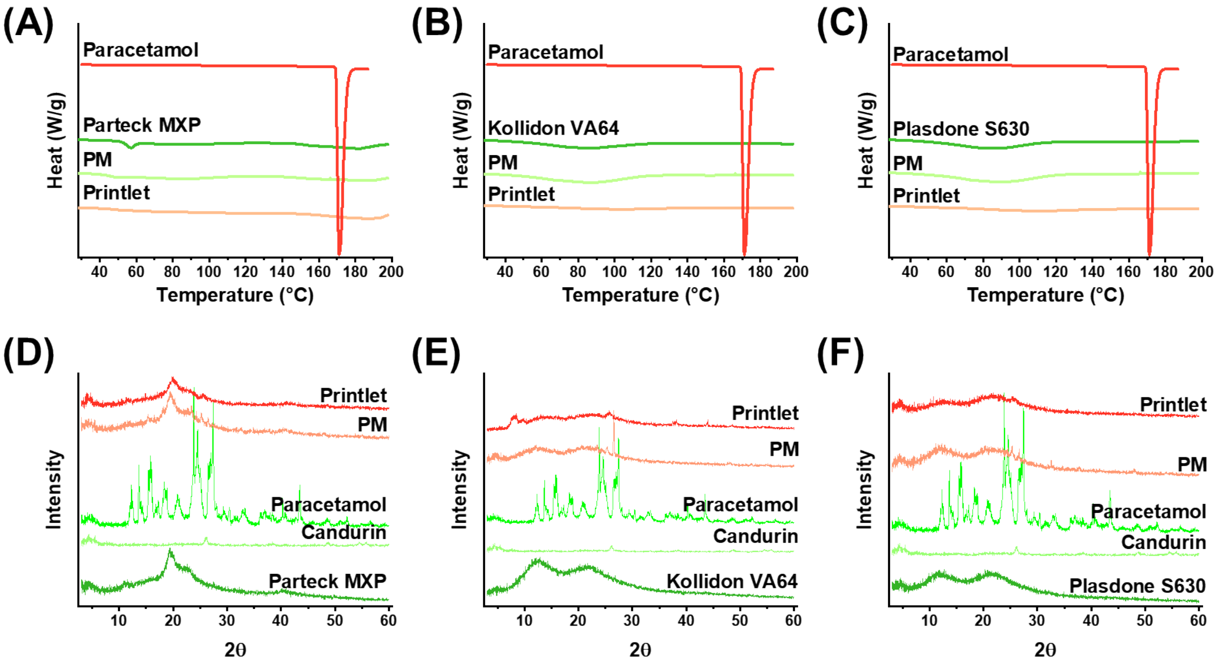

Figure S2. *(A)-(C) DSC and (D) – (F) XRD of the starting materials, their respective physical mixtures and the final printed product (printlet).*
